# Efficacy of tranilast in preventing exacerbating cardiac function and death from heart failure in muscular dystrophy patients with advanced-stage heart failure: a single-arm, open-label, multicenter study

**DOI:** 10.1186/s13023-025-03538-1

**Published:** 2025-01-09

**Authors:** Tsuyoshi Matsumura, Takayasu Fukudome, Yasufumi Motoyoshi, Akinori Nakamura, Satoshi Kuru, Kazuhiko Segawa, Ruriko Kitao, Chigusa Watanabe, Takuhisa Tamura, Toshiaki Takahashi, Hiroya Hashimoto, Masahiro Sekimizu, Akiko M. Saito, Masanori Asakura, Koichi Kimura, Yuko Iwata

**Affiliations:** 1 Department of Neurology, NHO Osaka Toneyama Medical Center, 5-1-1 Toneyama, Toyonaka, Osaka, 560-8552 Japan; 2 Department of Neurology, NHO Nagasaki Kawatana Medical Center, 2005-1 Shimogumigo, Kawatana, Nagasaki, 859-3615 Japan; 3 Department of Neurology, NHO Shimoshizu National Hospital, 934-5 Shikawatashi, Yotsukaido, Chiba, 284-0003 Japan; 4 Department of Clinical Research, NHO Matsumoto Medical Center, 2-20-30 Muraimachi-Minami, Matsumoto, Nagano, 399-8701 Japan; 5 Department of Neurology, NHO Suzuka Hospital, 3-2-1 Kasado, Suzuka, Mie, 513-8501 Japan; 6 Department of Cardiology, National Center of Neurology and Psychiatry, 4-1-1 Ogawahigashi, Kodaira, Tokyo, 187-8551 Japan; 7 Department of Neurology, NHO Hakone Hospital, 412 Kazamatsuri, Odawara, Kanagawa, 250-0032 Japan; 8 Department of Neurology, NHO Hiroshima-Nishi Medical Center, 4-1-1 Kuha, Otake, Hiroshima, 739-0696 Japan; 9 Department of Neurology, NHO Higashisaitama Hospital, 4147 Kurohama, Hasuda, Saitama, 349-0196 Japan; 10 Department of Neurology, NHO Sendai Nishitaga Hospital, 2-11-11 Kagitorihoncho, Taihaku-ku, Sendai, Miyagi, 982-8555 Japan; 11 Clinical Research Center, NHO Nagoya Medical Center, 4-1-1 Sannomaru, Naka-Ku, Nagoya, Aichi, 460-0001 Japan; 12 Department of Clinical Diagnosis and Laboratory Medicine, Hyogo College of Medicine, 1-1 Mukogawa, Nishinomiya, Hyogo, 663-8501 Japan; 13 Department of Laboratory Medicine/Cardiology, The Institute of Medical Science, The University of Tokyo, 4-6-1 Shirokanedai, Minato-ku, Tokyo, 108-8639 Japan; 14 Department of Cardiac Physiology, National Cerebral and Cardiovascular Center Research Institute, 6-1 Kishibe-Shimmachi, Suita, Osaka, 564-8565 Japan

**Keywords:** Transient receptor potential cation channel subfamily V member 2, Muscular dystrophy, Heart failure, Cardiac event, Tranilast

## Abstract

**Background:**

Transient receptor potential cation channel subfamily V member 2 (TRPV2) functions as a stretch-sensitive calcium channel, with overexpression in the sarcolemma of skeletal and cardiac myocytes leading to detrimental calcium influx and triggering muscle degeneration. In our previous pilot study, we showed that tranilast, a TRPV2 inhibitor, reduced brain natriuretic peptide levels in two patients with muscular dystrophy and advanced heart failure. Building on this, we performed a single-arm, open-label, multicenter study herein to evaluate the safety and efficacy of tranilast in the treatment of advanced heart failure in patients with muscular dystrophy.

**Results:**

This study involved 18 patients with muscular dystrophy who had brain natriuretic peptide levels > 100 pg/mL, despite receiving standard cardioprotective therapy. Tranilast was administered orally at a dose of 100 mg three times daily. Over the short-term period (28 weeks), the primary endpoint of change ratio in the logarithm of brain natriuretic peptide level from baseline to 28 weeks was not significant in the full analysis set but was lower in the per set protocol compared with data from a previous beta-blocker treatment study. All 15 patients who completed the short-term treatment consented to be enrolled in long-term therapy for an additional 116 weeks. After all participants completed the long-term treatment, we analyzed all data. TRPV2 expression on the peripheral blood mononuclear cell surfaces decreased throughout the study period, confirming that the TRPV2 inhibitory effect of tranilast was maintained over time. Despite the presence of progressive disease, cardiac indices such as brain natriuretic peptide level, human atrial natriuretic peptide level, and fractional shortening, remained stable, and only brain natriuretic peptide levels at 144 weeks showed significant changes. The survival rate was 80.7%, and no cardiac deaths were reported. Regarding safety, no serious adverse events associated with tranilast were noted, except for recurrent diarrhea during the short-term period in one case.

**Conclusions:**

The findings suggest that tranilast can inhibit TRPV2 expression for an extended period and is effective in preventing the worsening of cardiac function and subsequent death from heart failure.

**Clinical trial registration details:**

The study was registered in the UMIN Clinical Trials Registry (UMIN-CTR: UMIN000031965, URL: http://www.umin.ac.jp/ctr/) [March 30, 2018] and the Japan Registry of Clinical Trials (jRCT, registration number: jRCTs031180038, URL: https://jrct.niph.go.jp/) [November 12, 2021]. Patient registration was initiated on December 19, 2018.

**Supplementary Information:**

The online version contains supplementary material available at 10.1186/s13023-025-03538-1.

## Background

Muscular dystrophies (MDs) are a group of progressive and lethal diseases with no curative treatment. Formerly, respiratory failure and infections were the leading causes of death in MD patients; however, advancements in mechanical ventilation have markedly reduced the frequency of such respiratory deaths. Although widespread cardioprotective therapies, such as angiotensin-converting enzyme inhibitors (ACEI), angiotensin II receptor blockers (ARB), and beta-blockers, have improved outcomes for heart failure, their benefits have been insufficient. Currently, deaths due to MD are primarily caused by heart failure [1]. Although steroids are the primary recourse for treating MD, particularly Duchenne muscular dystrophy (DMD) [2], the exploration of novel therapies offers promising avenues for improving muscle function. Meanwhile, there is a concern that motor function improvement generated by new therapies may aggravate heart failure, underscoring the need for novel treatment options for heart failure.

One promising target, transient receptor potential cation channel subfamily V member 2 (TRPV2), is a stretch-sensitive calcium channel [3, 4]. Typically localized within intracellular membrane compartments, TRPV2 is translocated to the sarcolemma in damaged myocytes or cardiomyocytes. This translocated TRPV2 enhances calcium influx into the sarcolemma, triggering cell damage, and its overexpression in the sarcolemma has been observed in the cardiac and skeletal muscles of animal models of MD [4, 5]. TRPV2 overexpression in the sarcolemma has been observed in the skeletal muscle and cardiomyocytes of patients with MD [4, 5] and in the cardiomyocytes of patients with dilated cardiomyopathy (DCM) [5].

Previous research has reported that transgenic mice with cardiac-specific TRPV2 overexpression develop DCM-like symptoms [4]. Further, TRPV2 inhibition reportedly ameliorated the severity of muscle pathology, motor function, and cardiac function in various animal models of MDs and DCM [5–9]. We have previously developed a successful high-throughput screening method to detect compounds that inhibited TRPV2 [5, 9]. Tranilast, which has already been approved as a small-molecule anti-allergy drug, is one such drug. Tranilast has been reported to be effective in hamster models of cardiomyopathy and myocyte degeneration by removing TRPV2 from the sarcolemma of DCM-model hamsters [5, 6]. We previously performed a pilot study in which tranilast was used to treat two patients with MD and advanced cardiomyopathy. In both these patients, brain natriuretic peptide (BNP) levels decreased after tranilast therapy [10]. These patients also showed improved echocardiographic findings after more than a year of treatment [11].

Building on these promising outcomes, we subsequently conducted a single-arm, open-label, multicenter study to assess the efficacy of tranilast as an additional therapy for patients with MD and heart failure, with BNP levels > 100 pg/mL during standard cardiac protection therapy [12]. BNP is a standard cardiac function indicator that is highly correlated with the severity and prognosis of heart failure [13, 14]. In MD, a BNP level ≥ 100 pg/mL is considered a predictive risk factor for death [15]. The primary endpoint of the study was the logarithm of the BNP change ratio up to 28 weeks. In a previous multicenter study of carvedilol in which the null hypothesis was established, the mean population logarithm of the BNP change was 0.18 [16]. Since the BNP level in our study remained stable for 28 weeks, we rejected the null hypothesis in accordance with the study protocol [17].

This extensional study thus aimed to evaluate the long-term safety and efficacy of tranilast in patients who wished to continue tranilast after the initial 28 weeks, for an additional 116 weeks. Given the completion of the long-term treatment by all participants, we present a comprehensive analysis of our collected data.

## Methods

### Study design and participants

This single-arm, open-label, multicenter study aimed to evaluate the long-term safety and efficacy of tranilast in the treatment of heart failure in patients with MD. A full description of the study protocol was published in 2021 [12]. Briefly, the participants were patients with MD and advanced heart failure. The inclusion criteria were as follows: (1) age > 13 years, (2) plasma BNP level > 100 pg/mL at the time of enrollment, (3) receiving standard cardioprotective therapy, (4) without acute clinical status and severe arrhythmia, and (5) absence of renal/liver dysfunction and leukopenia. Tranilast was administered orally three times daily at 100 mg for 28 weeks (short-term treatment period). All patients who completed short-term treatment were eligible and consented to participate in long-term therapy (another 116 weeks).

### Outcomes

The primary endpoint was the change ratio in the logarithm of the BNP level at 28 weeks. The secondary endpoints were cardiac events; total death; and adverse events including changes in cardiac indices [fractional shortening (FS), human atrial natriuretic peptide (hANP), cardiac troponin T (cTnT), and BNP], creatine kinase (CK), grip strength, Muscular Dystrophy Quality of Life-60 (MDQoL-60), the 12-item Short Form Health Survey (SF-12) score, and expression level of TRPV2 at the cellular membrane of peripheral mononuclear cells.

The primary endpoint data were already reported previously [17]; therefore, we analyzed all above-mentioned evaluation parameters at 144 weeks in the present study. Given that this was a single-arm study, no control data were available. For reference, we collected data from patients with dystrophinopathies who had BNP levels > 100 pg/mL in 2019 at the NHO Osaka Toneyama Medical Center.

### Statistical analysis

We set a target number of 20 patients to demonstrate superiority of the change from baseline in log BNP values at 28 weeks compared to that in the historical control [12]. Patient characteristics are presented as mean and standard deviation for continuous data following a normal distribution, as median and interquartile range for other continuous data, and as frequencies and percentages for nominal data, and were compared using the two-sample t test, Wilcoxon rank-sum test, and Fisher’s exact test, respectively. For the time to onset of cardiac events, cumulative incidence was estimated using all-cause mortality as the competing risk. Survival curves were estimated using the Kaplan–Meier method and compared using the log-rank test. Repeated measures continuous data were estimated with the mean change from baseline and its 95% confidence interval (CI). However, since BNP, hANP, and cTnT were considered to follow a lognormal distribution, the percent change based on the geometric mean and its CI were estimated. All statistical analyses were performed using SAS version 9.4 (SAS Institute, Cary, NC, USA). A *p*-value of < 0.05 was considered statistically significant.

### Ethics

The National Hospital Organization Review Board and the Advanced Medical Technology of Health, Labour, and Welfare Ministry panel approved the Clinical Trials protocol. All patients provided written informed consent. The study was registered in the UMIN Clinical Trials Registry (UMIN-CTR: UMIN000031965, URL: http://www.umin.ac.jp/ctr/) [March 30, 2018] and the Japan Registry of Clinical Trials (jRCT, registration number: jRCTs031180038, URL: https://jrct.niph.go.jp/) [November 12, 2021].

The protocol for collecting reference data was approved by the Ethical Review Board of NHO Osaka Toneyama Medical Center (approval number: TNH-R-2023031). In this retrospective study, we disclosed the information and guaranteed opt-out opportunities.

## Results

### Participants

**Fig. 1:**
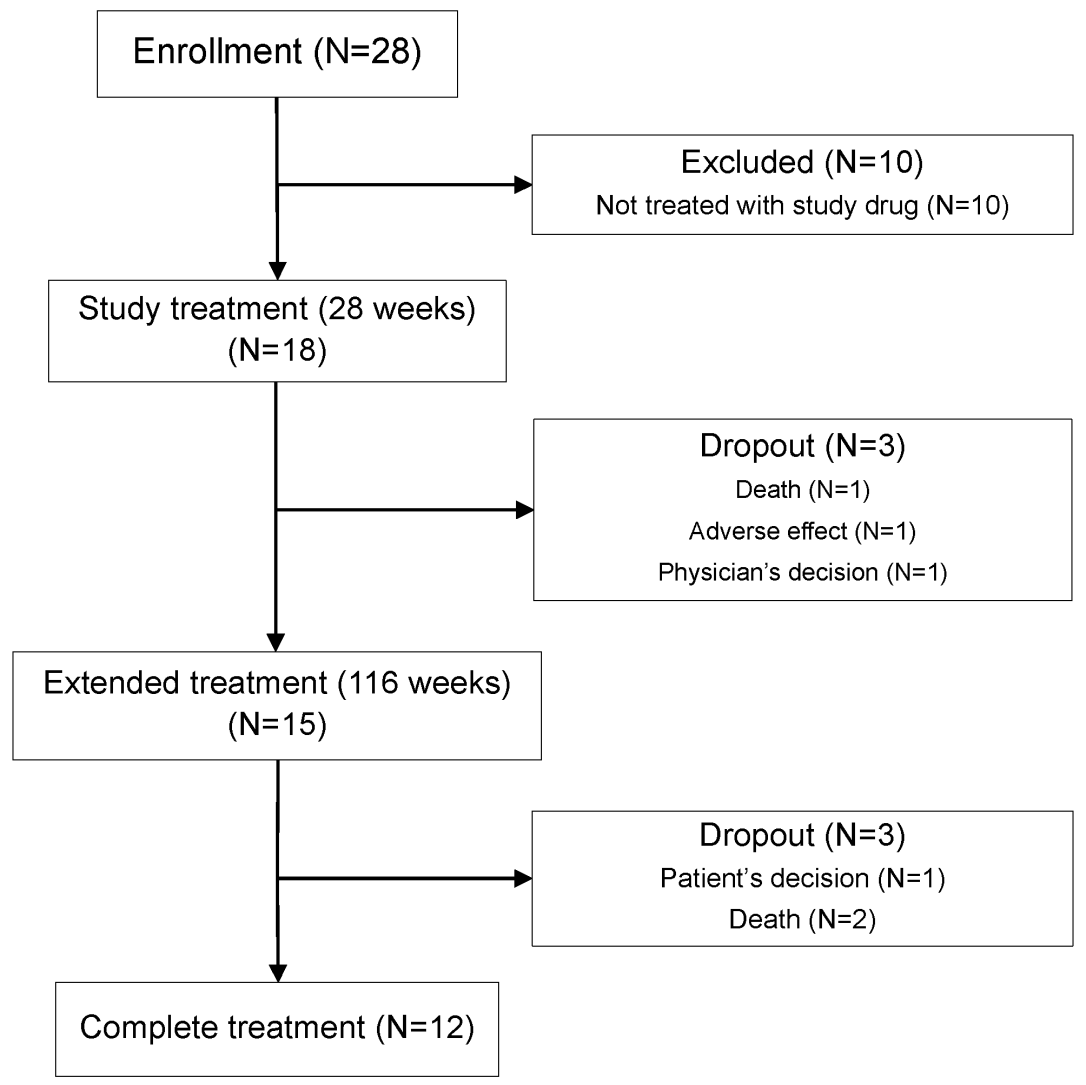
Flow chart of the study and patient enrollment

We enrolled 28 patients in the study, introduced the study treatment to 18 patients, and collected valid data after treatment from 17 patients (DMD: 13, Becker muscular dystrophy (BMD): 3, α-sarcoglycanopathy: 1). One patient with DMD died of sepsis during the short-term treatment period (within 28 weeks). Another patient with DMD discontinued treatment because of recurrent diarrhea. Consequently, 15 patients completed the short-term treatment [17]. All 15 participants wished to continue tranilast therapy. However, two patients with DMD died and one with BMD withdrew from the study. As a result, 12 patients completed the long-term study (Fig. 1).

### Safety

**Fig. 2:**
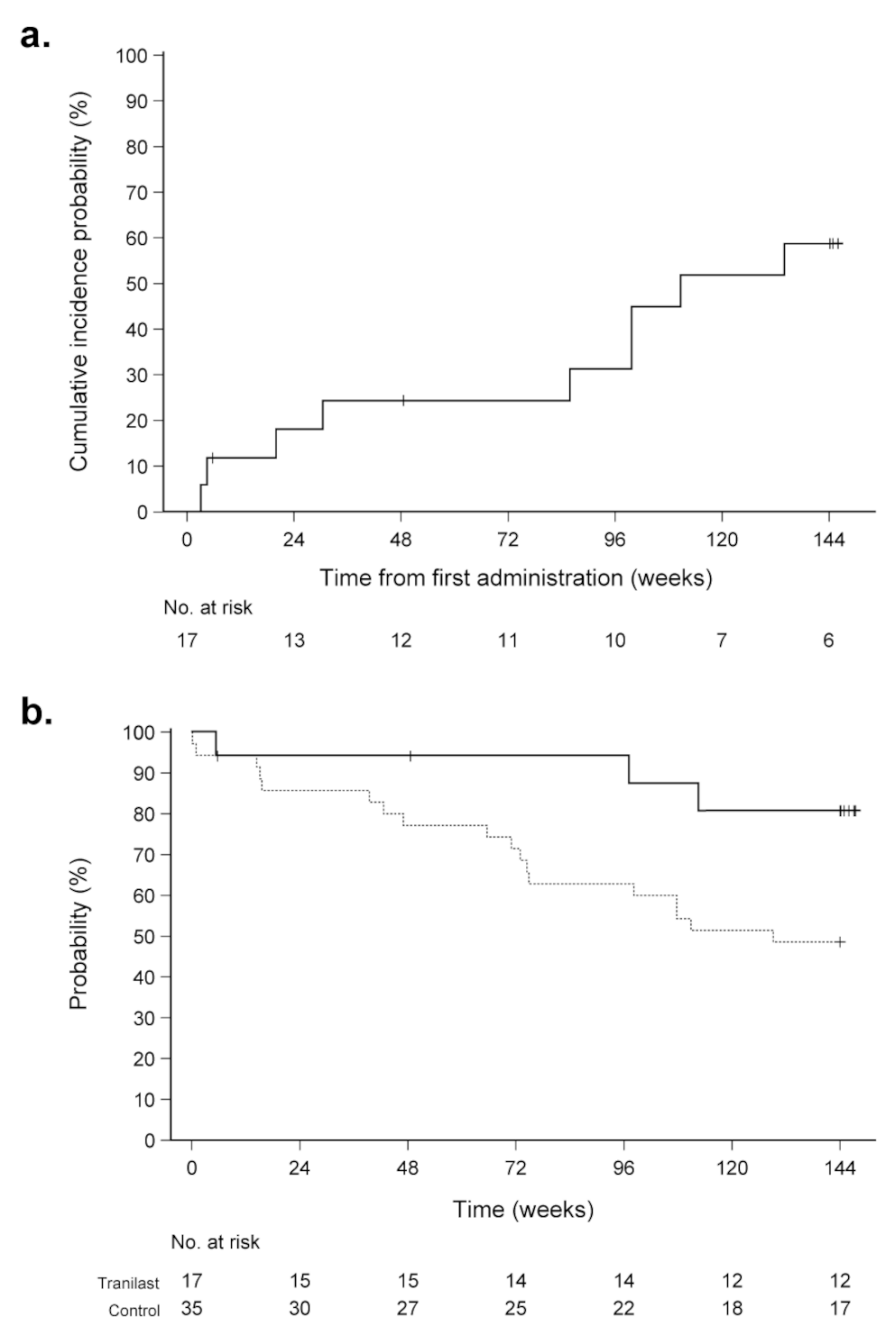
Cumulative rate of cardiac events and survival probability of patients undergoing long-term tranilast treatment. (**a**) Cumulative probability of incidence of cardiac events among patients undergoing tranilast treatment. (**b**) Survival probability of patients undergoing tranilast treatment compared to reference data from our hospital Solid line: tranilast treatment group, dashed line: reference data

Nine cardiac events occurred during the entire treatment period, with a cumulative rate of 58.7%. Of these, three required intravenous medications [severe adverse events (SAEs)]: one for exacerbating cardiac failure due to non-steroid anti-inflammatory drug administration for pain and two for septic shock from aspiration pneumonia. Six patients changed their oral medications: one each for increased pleural effusion, asymptomatic elevation of BNP (SAE), increased arrhythmia, digitalis discontinuation due to atrioventricular block, progressive renal dysfunction due to increased diuretics administered before study entry (SAE) [17], and change in prescription dose due to miscommunication. The cumulative rates of the cardiac events are shown in Fig. 2a.

A total of 11 SAEs occurred in 8 patients: 3 deaths [2 developed sepsis due to aspiration pneumonia (as described above) and 1 choked]. Other SAEs included congestive heart failure triggered by pneumonia, hospitalization due to electrolyte abnormalities, and increased arrhythmias associated with dehydration caused by heat shock, gastrostomy due to swallowing dysfunction, appendicitis, and recurrent diarrhea (as described above).

### Survival rate

The 144-week survival rate in this study was 80.7%. None of the patients died of heart failure. As this was an open-label, single-arm study, no control data were available. Although this bias cannot be ruled out, we compared the results with those of 35 patients with dystrophinopathy (26 with DMD, 6 with BMD, and 3 carriers), who showed BNP levels > 100 pg/mL at the NHO Osaka Toneyama Medical Center in 2019. The average age and BNP levels of these patients were comparable to those of the participants, and their average FS was better than that of the participants (Table 1). Nonetheless, 18 patients died within 144 weeks (13 with DMD, 4 with BMD, and 1 carrier, including 11 deaths due to heart failure). The log-rank test using the Kaplan–Meier method showed *p*-values of 0.039 (Fig. 2b).

**Table 1:** Profiles of tranilast treated and untreated patients

	Tranilast treated	Tranilast untreated	*p*-value
Disease			
DMD	13 (72.2%)	26 (74.3%)	0.213
BMD	3 (16.7%)	6 (17.1%)	
Carrier	0	3 (8.6%)	
LGMD	2 (11.1%) 1: α-sarcoglycanopathy 1: unclassified LGMD	0	
Sex: Male/Female	16/2	32/3	1.000
Age: Average ± SD	36.8 ± 15.6	39.4 ± 15.9	0.582
BNP: Median (IQR)	185 (132–260)	142 (109–245)	0.430
FS: Average ± SD	10.2 ± 6.4	15.4 ± 7.8 (*n* = 23)	0.014

^a^Abbreviations: SD: standard deviation, IQR: interquartile range, DMD: Duchenne muscular dystrophy, BMD: Becker muscular dystrophy, BNP: brain natriuretic peptide, FS: fractional shortening, LGMD: limb-girdle muscular dystrophy

### Cardiac function

**Fig. 3:**
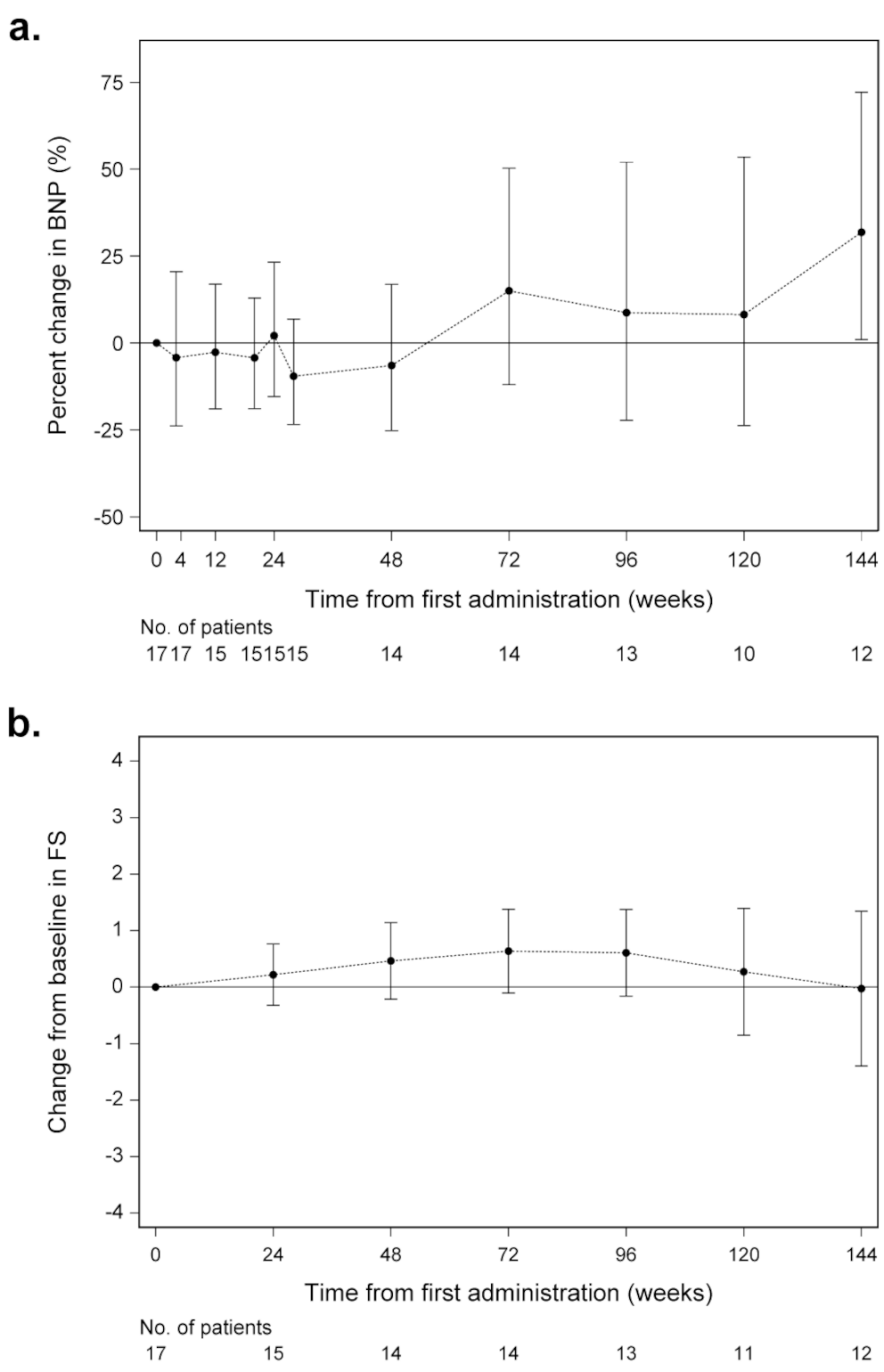
Changes in cardiac functions in patients undergoing long-term tranilast treatment. (**a**) Percentage change in log-transformed brain natriuretic peptide (BNP) levels. (**b**) Changes in fractional shortening (FS) from baseline Error bar: 95% CI (confidence interval)

The geometric mean (GM) BNP levels were maintained for 48 weeks. Subsequently, there was an increasing trend, but the 95% CI for the rate of change from baseline included 0, except at 144 weeks (Fig. 3a). The GM of hANP also maintained baseline values until 48 weeks and then showed an increasing trend. However, the 95% CI of the rate of change from baseline included 0 for all periods (Additional file 1: Table S1). The GM of cTnT increased from 0.026 at baseline to 0.030 after the first 4 weeks of treatment but remained almost unchanged during the overall study period. The 95% CI for the rate of change from baseline included 0 for all periods except at 24 weeks (Additional file 1: Table S2).

Echocardiographic findings showed that FS exceeded baseline for up to 72 weeks after the start of treatment, which was when the trend began decreasing. However, the 95% CI for the change from baseline included 0 at all time points (Fig. 3b).

Holter electrocardiography showed a slight downward trend in the mean heart rate. The total number of premature ventricular contractions mildly increased for up to 48 weeks, followed by a downward trend (Additional file 1: Table S3).

### TRPV2 expression in peripheral blood mononuclear cell surface

**Fig. 4:**
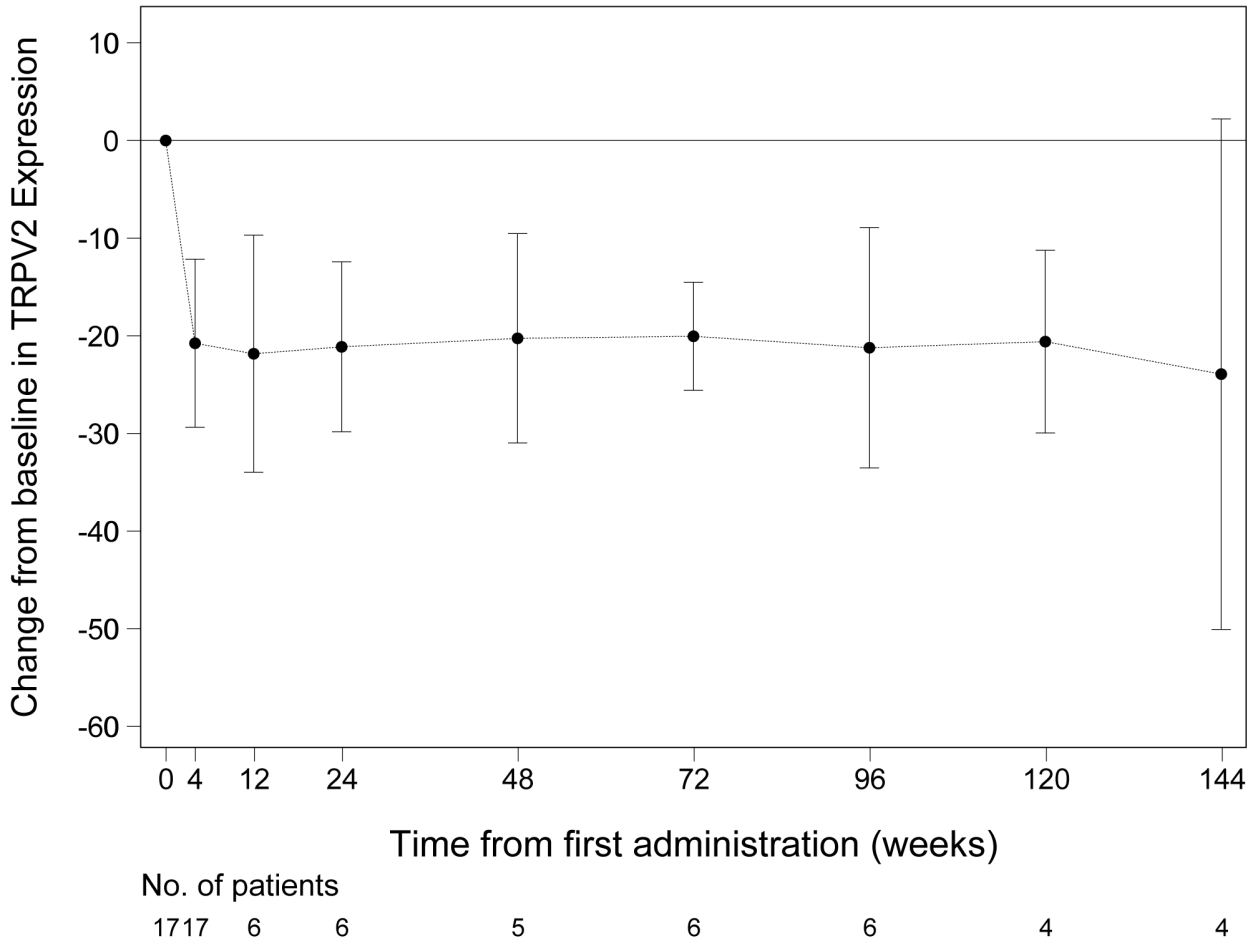
Changes in membrane TRPV2 expression in peripheral mononuclear cells after long-term tranilast treatment Error bar: 95% CI (confidence interval)

This analysis was an essential item before and after 4 weeks of treatment and an effort item after 12 weeks. Therefore, we retrieved the data of only 4–6 patients after the 12-week mark. However, all patients maintained lower levels after treatment throughout the study (Fig. 4).

### Skeletal muscle indices

We evaluated the CK levels and pinch force as skeletal muscle indices. As the participants in this study had advanced diseases, the median CK level was 221 U/L (interquartile range, IQR: 152–284 U/L). Although no significant changes were observed after the treatment, a downward trend was observed after 48 weeks. The median pinch force was as low as 0.40 N (IQR: 0.2–0.6) at baseline. Although a slight decreasing trend was observed, no significant differences were detected (Additional file 1: Table S4).

### Renal function

One patient showed progressive renal dysfunction in the short-term treatment period due to increased diuretics administered before study enrollment (described above). The details of this patient have been reported in a previous paper [17]. Except for this single case, no serious adverse events were associated with renal dysfunction. Although renal function values showed an increasing trend over time, the differences were not significant (Additional file 1: Table S5).

### Self-questionnaire of quality of life

No MDQoL-60 items, except sex, showed obvious changes at 48 weeks (Additional file 1: Table S6). SF-12 showed no apparent changes, except for role-emotional article at 24 weeks (Additional file 1: Table S7).

## Discussion

In this study, we examined the safety and efficacy of tranilast in treating heart failure in patients with MD. The results of this study suggested that tranilast has a long-term, stable TRPV2 inhibitory effect that prevents worsening of cardiac function and cardiac death and can be administered relatively safely even to patients with MD and advanced heart failure.

First, we validated the long-term effect of tranilast in inhibiting TRPV2. TRPV2 expression in peripheral blood mononuclear cell surface reduced significantly after initiating tranilast. Although the sample size of our study was limited, all participants maintained lower levels throughout the study.

Second, our data suggested that tranilast prevents the progression of cardiac dysfunction and cardiac death. No participants in the present study showed drastic improvement, as seen in the pilot study [10]. However, the primary endpoint, log BNP change up to 28 weeks, rejected the null hypothesis based on data from a previous beta-blocker study on PPS [17]. During long-term treatment, crucial cardiac function indices—such as FS, BNP, and hANP—remained stable despite progressive disease. Furthermore, there were no reported deaths due to heart failure among patients receiving the study treatment, and the 144-week survival rate exceeded 80%. We could not perform a direct comparison because we did not include a control group. However, the 144-week survival rate of 35 patients with dystrophinopathy, whose BNP level exceeded 100 pg/mL in 2019, was approximately 50% at our hospital (*p* = 0.046). Additionally, 11 patients died of heart failure. All these patients received standard cardioprotective care, and the FS of the untreated reference group was significantly higher than that of the treated participants.

Third, tranilast was considered relatively safe for these participants. Given that the participants in this study were patients with MD and advanced heart failure, their general condition was poor. We noted 11 SAEs and 3 death events during the entire study period. However, a causal relationship with tranilast was suggested in only one case of recurrent diarrhea. No SAEs were associated with tranilast during long-term treatment or other adverse events that could interfere with continued treatment. Based on the findings of the pilot study, we closely monitored arrhythmia and renal function. However, we did not detect any tranilast-associated events. Although a slightly increasing trend in renal indices was observed, this phenomenon was due to cardiorenal syndrome, which is naturally observed in patients with MD and advanced heart failure [18, 19].

This study has some limitations, including the unstable general condition of the participants, small sample size, and lack of a control group. We focused on the treatment of heart failure and therefore had to target patients with advanced conditions who did not respond adequately to standard therapy. The instability of patients’ general condition affected the assessment of cardiac function, making skeletal muscle assessment difficult. Based on the pharmacological mechanism of TRPV2 inhibitor therapy, the appropriate timing for starting administration should be reconsidered. TRPV2 overexpression at the sarcolemma level in damaged myocytes and cardiomyocytes can induce intracellular calcium influx, triggering a degenerating process [4–9]. Recent data indicate that TRPV2 may involve macrophage-dependent pro-inflammatory processes and cardiac fibrosis [20, 21]. These facts suggest that tranilast should be introduced during the active skeletal and cardiac degeneration phase.

Another limitation is the absence of a control group and small sample size. At the time of this study, there were no other trials of tranilast as a treatment for heart failure other than our pilot study. Therefore, as a “proof of concept” study, we were instructed to conduct this study with an open-label, single-arm approach in a small number of patients. We compared the primary endpoint with data from previous trials and the survival rate with data from our hospital; however, the lack of a control group made exact comparisons difficult.

To overcome these limitations, we are launching a placebo-controlled, randomized, double-blind study targeting DMD patients with active skeletal and cardiac degeneration, which is designed to evaluate the effects on motor, respiratory, and cardiac function. We hope that subsequent studies will clarify the efficacy of tranilast for MD.

## Conclusions

Our data suggest that TRPV2 inhibitor therapy may be effective in patients with MD and advanced heart failure. However, to comprehensively evaluate efficacy in skeletal muscle and myocardium, clinical trials targeting early-stage patients are imperative. This research paves the way for future investigations into the preventive potential of tranilast in preventing the progression of skeletal and cardiac muscle damage.

## Electronic supplementary material

Below is the link to the electronic supplementary material.

Additional file 1: Supplementary Tables S1–S7

## Data Availability

The datasets used and analyzed during the current study are not openly available to protect study participant privacy and are available from the corresponding author on reasonable request. Data are located in controlled access data storage at NHO Nagoya Medical Center https://nagoya.hosp.go.jp/crc/clinical_trial_services/crb/.

## Abbreviations

ACEI: Angiotensin converting enzyme inhibitors
ARB: Angiotensin II receptor blockers
hANP: Human atrial natriuretic peptide
BNP: Brain natriuretic peptide
CI: Confidence interval
CK: Creatine kinase
cTnT: Cardiac troponin T
DCM: Dilated cardiomyopathy
DMD: Duchenne muscular dystrophy
FS: Fractional shortening
GM: Geometric mean
MD: Muscular dystrophy
MDQoL-60: Muscular dystrophy quality of life-60
SAE: Severe adverse events
SF-12: The 12-item Short Form Health Survey
TRPV2: Transient receptor potential cation channel subfamily V member 2

## References

[1] Matsumura T, Saito T, Fujimura H, Shinno S, Sakoda S. A longitudinal cause-of-death analysis of patients with Duchenne muscular dystrophy. Rinsho Shinkeigaku. 2011;51. 10.5692/clinicalneurol.51.743. 743 – 50 [Japanese]. 10.5692/clinicalneurol.51.743 22019865

[2] Birnkrant DJ, Bushby K, Bann CM, Apkon SD, Blackwell A, Brumbaugh D, et al. Diagnosis and management of Duchenne muscular dystrophy, part 1: diagnosis, and neuromuscular, rehabilitation, endocrine, and gastrointestinal and nutritional management. Lancet Neurol. 2018;17:251–67. 10.1016/s1474-4422(18)30024-3 29395989 10.1016/S1474-4422(18)30024-3 PMC5869704

[3] Muraki K, Iwata Y, Katanosaka Y, Ito T, Ohya S, Shigekawa M, et al. TRPV2 is a component of osmotically sensitive cation channels in murine aortic myocytes. Circ Res. 2003;93:829–38. 10.1161/01.RES.0000097263.10220.0C 14512441 10.1161/01.RES.0000097263.10220.0C

[4] Iwata Y, Katanosaka Y, Arai Y, Komamura K, Miyatake K, Shigekawa M. A novel mechanism of myocyte degeneration involving the Ca2+-permeable growth factor–regulated channel. J Cell Biol. 2003;161:957–67. 10.1083/jcb.200301101 12796481 10.1083/jcb.200301101 PMC2172975

[5] Iwata Y, Ohtake H, Suzuki O, Matsuda J, Komamura K, Wakabayashi S. Blockade of sarcolemmal TRPV2 accumulation inhibits progression of dilated cardiomyopathy. Cardiovasc Res. 2013;99:760–8. 10.1093/cvr/cvt163 23786999 10.1093/cvr/cvt163

[6] Iwata Y, Katanosaka Y, Shijun Z, Kobayashi Y, Hanada H, Shigekawa M, et al. Protective effects of Ca2 + handling drugs against abnormal Ca2 + homeostasis and cell damage in myopathic skeletal muscle cells. Biochem Pharmacol. 2005;70:740–51. 10.1016/j.bcp.2005.05.034 16009351 10.1016/j.bcp.2005.05.034

[7] Iwata Y, Katanosaka Y, Arai Y, Shigekawa M, Wakabayashi S. Dominant-negative inhibition of Ca2 + influx via TRPV2 ameliorates muscular dystrophy in animal models. Hum Mol Genet. 2009;18:824–34. 10.1093/hmg/ddn408 19050039 10.1093/hmg/ddn408

[8] Zanou N, Iwata Y, Schakman O, Lebacq J, Wakabayashi S, Gailly P. Essential role of TRPV2 ion channel in the sensitivity of dystrophic muscle to eccentric contractions. FEBS Lett. 2009;583:3600–4. 10.1016/j.febslet.2009.10.033 19840792 10.1016/j.febslet.2009.10.033

[9] Iwata Y, Katayama Y, Okuno Y, Wakabayashi S. Novel inhibitor candidates of TRPV2 prevent damage of dystrophic myocytes and ameliorate against dilated cardiomyopathy in a hamster model. Oncotarget. 2018;9:14042–57. 10.18632/oncotarget.24449 29581825 10.18632/oncotarget.24449 PMC5865651

[10] Matsumura T, Matsui M, Iwata Y, Asakura M, Saito T, Fujimura H, et al. A pilot study of tranilast for cardiomyopathy of muscular dystrophy. Intern Med. 2018;57:311–8. 10.2169/internalmedicine.8651-16 29093384 10.2169/internalmedicine.8651-16 PMC5827307

[11] Iwata Y, Matsumura T. Blockade of TRPV2 is a novel therapy for cardiomyopathy in muscular dystrophy. Int J Mol Sci. 2019;20:3844. 10.3390/ijms20163844 31394715 10.3390/ijms20163844 PMC6720432

[12] Matsumura T, Hashimoto H, Sekimizu M, Saito AM, Iwata Y, Asakura M, et al. Study protocol for a multicenter, open-label, single-arm study of tranilast for cardiomyopathy of muscular dystrophy. Kurume Med J. 2021;66:121–6. 10.2739/kurumemedj.MS662006 34135201 10.2739/kurumemedj.MS662006

[13] Tsutsui H, Albert NM, Coats AJS, Anker SD, Bayes-Genis A, Butler J, et al. Natriuretic peptides: role in the diagnosis and management of heart failure: a scientific statement from the Heart Failure Association of the European Society of Cardiology, Heart Failure Society of America and Japanese Heart failure society. Eur J Heart Fail. 2023;25:616–31. 10.1002/ejhf.2848 37098791 10.1002/ejhf.2848

[14] Nikhanj A, Nichols BM, Wang K, Siddiqi ZA, Oudit GY. Evaluating the diagnostic and prognostic value of biomarkers for heart disease and major adverse cardiac events in patients with muscular dystrophy. Eur Heart J Qual Care Clin Outcomes. 2021;7:564–73. 10.1093/ehjqcco/qcaa059 32687175 10.1093/ehjqcco/qcaa059

[15] Demachi J, Kagaya Y, Watanabe J, Sakuma M, Ikeda J, Kakuta Y, et al. Characteristics of the increase in plasma brain natriuretic peptide level in left ventricular systolic dysfunction, associated with muscular dystrophy in comparison with idiopathic dilated cardiomyopathy. Neuromuscul Disord. 2004;14:732–9. 10.1016/j.nmd.2004.08.002 15482958 10.1016/j.nmd.2004.08.002

[16] Matsumura T, Tamura T, Kuru S, Kikuchi Y, Kawai M. Carvedilol can prevent cardiac events in Duchenne muscular dystrophy. Intern Med. 2010;49:1357–63. 10.2169/internalmedicine.49.3259 20647648 10.2169/internalmedicine.49.3259

[17] Matsumura T, Hashimoto H, Sekimizu M, Saito AM, Motoyoshi Y, Nakamura A, et al. Tranilast for advanced heart failure in patients with muscular dystrophy: a single-arm, open-label, multicenter study. Orphanet J Rare Dis. 2022;17:201. 10.1186/s13023-022-02352-3 35578298 10.1186/s13023-022-02352-3 PMC9109199

[18] Matsumura T, Saito T, Fujimura H, Sakoda S. Renal dysfunction is a frequent complication in patients with advanced stage of Duchenne muscular dystrophy. Rinsho Shinkeigaku. 2012;52:211–7. 10.5692/clinicalneurol.52.211. [Japanese]. 22531652 10.5692/clinicalneurol.52.211

[19] Motoki T, Shimizu-Motohashi Y, Saito I, Komaki H, Ishiyama A, Aibara K, et al. Renal dysfunction can occur in advanced-stage Duchenne muscular dystrophy. Muscle Nerve. 2020;61:192–7. 10.1002/mus.26757 31725904 10.1002/mus.26757

[20] Entin-Meer M, Cohen L, Hertzberg-Bigelman E, Levy R, Ben-Shoshan J, Keren G. TRPV2 knockout mice demonstrate an improved cardiac performance following myocardial infarction due to attenuated activity of peri-infarct macrophages. PLoS ONE. 2017;12:e0177132. 10.1371/journal.pone.0177132 28481959 10.1371/journal.pone.0177132 PMC5421795

[21] Feng J, Armillei MK, Yu AS, Liang BT, Runnels LW, Yue L. Ca2 + signaling in cardiac fibroblasts and fibrosis-associated heart diseases. J Cardiovasc Dev Dis. 2019;6:34. 10.3390/jcdd6040034 31547577 10.3390/jcdd6040034 PMC6956282

