# Supplementary material for: Efficacy of tranilast in preventing exacerbating cardiac function and death from heart failure in muscular dystrophy patients with advanced-stage heart failure: a single-arm, open-label, multicenter study
Source: Orphanet J Rare Dis. 2025 Jan 9;20:13. doi: 10.1186/s13023-025-03538-1 (PMC11720297; doi:10.1186/s13023-025-03538-1)
Supplement: Supplementary file 1 — Additional file 1: Supplementary Tables S1–S7 [file 13023_2025_3538_MOESM1_ESM.docx]

**Supplementary Materials**

**Supplementary Table S1.** **Atrial natriuretic peptide data.**

|  | n | GM | GSD | Median | IQR | Range | Change ratio (%) Point estimation (95% CI) | P value* |
| --- | --- | --- | --- | --- | --- | --- | --- | --- |
| 0 week | 17 | 189.7 | 1.74 | 223.0 | 152–284 | 77–378 |  |  |
| 4 weeks | 17 | 175.6 | 2.04 | 214.0 | 102–257 | 44–515 | -7.4 (-21.8, 9.6) |  |
| 12 weeks | 15 | 193.7 | 1.73 | 216.0 | 114–315 | 77–370 | 0.4 (-16.2, 20.3) |  |
| 24 weeks | 15 | 206.8 | 1.99 | 257.0 | 123–380 | 55–445 | 7.2 (-12.1, 30.8) |  |
| 24 weeks or at termination** | 17 | 199.1 | 2.19 | 257.0 | 123–380 | 44–515 | 5.0 (-13.6, 27.6) | 0.605 |
| 48 weeks | 15 | 199.1 | 1.96 | 224.0 | 101–305 | 63–572 | 3.2 (-19.5, 32.3) |  |
| 72 weeks | 14 | 220.3 | 2.10 | 235.5 | 125–370 | 45–633 | 13.4 (-10.0, 43.0) |  |
| 96 weeks | 13 | 237.0 | 2.32 | 254.0 | 158–392 | 43–970 | 19.8 (-11.4, 61.8) |  |
| 120 weeks | 10 | 248.3 | 2.60 | 278.0 | 97–587 | 57–1040 | 36.2 (-6.7, 98.9) |  |
| 144 weeks | 12 | 251.7 | 2.54 | 295.5 | 96–454 | 54–1160 | 28.7 (-13.7, 91.9) |  |

Abbreviations: n: number, GM: geometric mean, GSD: geometric standard deviation, IQR: interquartile range, CI: confidence interval

** We performed t-tests for the log-transformed change on values at 24 weeks or discontinuation up to 24 weeks.

**Supplementary Table S2.** **Cardiac troponin T data.**

|  | n | GM | GSD | Median | IQR | Range | Change ratio (%) Point estimation (95% CI) | P value* |
| --- | --- | --- | --- | --- | --- | --- | --- | --- |
| 0 week | 17 | 0.026 | 1.76 | 0.024 | 0.02–0.03 | 0.01–0.08 |  |  |
| 4 weeks | 17 | 0.030 | 1.79 | 0.026 | 0.02–0.05 | 0.01–0.10 | 12.5 (-1.5, 28.5) |  |
| 12 weeks | 15 | 0.030 | 1.99 | 0.027 | 0.02–0.06 | 0.01–0.11 | 13.5 (0.0, 28.7) |  |
| 24 weeks | 15 | 0.030 | 1.93 | 0.022 | 0.02–0.05 | 0.01–0.14 | 12.0 (0.2, 25.1) |  |
| 24 weeks or at termination** | 17 | 0.030 | 1.88 | 0.022 | 0.02–0.05 | 0.01–0.14 | 14.6 (1.1, 29.9) | 0.035 |
| 48 weeks | 15 | 0.031 | 2.02 | 0.024 | 0.02–0.05 | 0.01–0.14 | 17.9 (-5.2, 46.5) |  |
| 72 weeks | 14 | 0.029 | 1.79 | 0.026 | 0.02–0.03 | 0.01–0.09 | 12.9 (-6.1, 35.8) |  |
| 96 weeks | 13 | 0.031 | 1.70 | 0.028 | 0.03–0.04 | 0.01–0.10 | 15.7 (-1.8, 36.4) |  |
| 120 weeks | 10 | 0.031 | 1.70 | 0.030 | 0.02–0.05 | 0.01–0.08 | 9.2 (-10.9, 33.9) |  |
| 144 weeks | 12 | 0.032 | 1.81 | 0.029 | 0.02–0.05 | 0.01–0.10 | 17.0 (-4.3, 43.2) |  |

Abbreviations: n: number, GM: geometric mean, GSD: geometric standard deviation, IQR: interquartile range, CI: confidence interval

** We performed t-tests for the log-transformed change on values at 24 weeks or discontinuation up to 24 weeks.

**Supplementary Table S3.** **Holter Electrocardiogram data.**

| Items | n | Mean | SD | Median | IQR | Range |
| --- | --- | --- | --- | --- | --- | --- |
| Mean heart rate (beats/minute) |  |  |  |  |  |  |
| Pre-treatment | 18 | 80.4 | 12.6 | 79.0 | 73–90 | 58–103 |
| 24 weeks | 16 | 80.8 | 12.4 | 80.5 | 72–89 | 60–114 |
| 48 weeks | 14 | 78.4 | 12.2 | 74.5 | 71–86 | 60–100 |
| 72 weeks | 15 | 77.4 | 8.7 | 75.0 | 72–82 | 63–96 |
| 96 weeks | 12 | 74.3 | 9.9 | 75.5 | 64–82 | 62–89 |
| 120 weeks | 12 | 76.8 | 9.7 | 78.0 | 70–85 | 61–89 |
| 144 weeks | 12 | 76.1 | 10.5 | 74.0 | 70–81 | 59–95 |
| Premature ventricular contraction (beats/day) |  |  |  |  |  |  |
| Pre-treatment | 18 | 694.9 | 1280.0 | 187.0 | 57–657 | 1–5026 |
| 24 weeks | 16 | 1489.1 | 2648.5 | 268.0 | 99–1879 | 1–10169 |
| 48 weeks | 14 | 1606.1 | 2584.7 | 514.0 | 159–1882 | 6–9307 |
| 72 weeks | 15 | 1517.4 | 2986.2 | 475.0 | 168–1295 | 26–11766 |
| 96 weeks | 12 | 1498.2 | 2268.7 | 401.0 | 99–2006 | 38–6145 |
| 120 weeks | 12 | 1111.3 | 1997.9 | 256.0 | 104–626 | 21–5780 |
| 144 weeks | 12 | 865.7 | 1294.0 | 360.0 | 146–1152 | 11–4698 |

Abbreviations: n: number, SD: standard deviation, IQR: interquartile range

**Supplementary Table S4.** **Creatine kinase data.**

|  | n | GM | GSD | Median | IQR | Range | Change ratio (%) Point estimation (95% CI) | P value* |
| --- | --- | --- | --- | --- | --- | --- | --- | --- |
| 0 week | 17 | 269 | 2.12 | 221 | 157–382 | 88–1996 |  |  |
| 4 weeks | 17 | 298 | 2.42 | 256 | 166–519 | 50–2651 | 10.8 (-13.6, 42.0) |  |
| 12 weeks | 15 | 295 | 2.19 | 279 | 146–414 | 97–1964 | 3.2 (-10.3, 18.8) |  |
| 20 weeks | 15 | 308 | 2.40 | 245 | 177–447 | 105–3497 | 7.7 (-11.2, 30.7) |  |
| 24 weeks | 15 | 322 | 2.05 | 293 | 169–478 | 132–1668 | 12.5 (-13.2, 45.7) |  |
| 24 weeks or at termination** | 17 | 299 | 2.29 | 293 | 169–478 | 50–1668 | 11.2 (-16.4, 47.9) | 0.441 |
| 28 weeks | 15 | 272 | 2.22 | 226 | 158–448 | 81–2014 | -5.1 (-20.3, 12.9) |  |
| 48 weeks | 14 | 222 | 1.80 | 197 | 161–334 | 86–593 | -10.9 (-26.3, 7.6) |  |
| 72 weeks | 14 | 234 | 1.94 | 199 | 141–364 | 89–956 | -6.2 (-21.3, 11.7) |  |
| 98 weeks | 13 | 242 | 1.83 | 226 | 177–427 | 86–541 | -10.3 (-28.0, 11.7) |  |
| 120 weeks | 11 | 239 | 1.77 | 212 | 139–385 | 103–621 | -21.0 (-43.7, 10.7) |  |
| 144 weeks | 12 | 219 | 1.84 | 246 | 123–360 | 88–609 | -22.6 (-42.2, 3.7) |  |

Abbreviations: n: number, GM: geometric mean, GSD: geometric standard deviation, IQR: interquartile range, CI: confidence interval

** We performed t-tests for the log-transformed change on values at 24 weeks or discontinuation up to 24 weeks.

**Supplementary Table S5.** **Renal data.**

| Items | n | Mean | SD | Median | IQR | Range |
| --- | --- | --- | --- | --- | --- | --- |
| BUN (mg/dL) |  |  |  |  |  |  |
| 0 week | 18 | 14.49 | 6.74 | 13.20 | 9.9–17.5 | 5.1–34.5 |
| 4 weeks | 18 | 18.22 | 10.27 | 14.25 | 12.1–22.8 | 7.3–41.2 |
| 12 weeks | 16 | 18.54 | 12.63 | 15.25 | 11.0–18.9 | 7.8–54.5 |
| 24 weeks | 16 | 17.17 | 11.15 | 13.85 | 11.4–18.8 | 5.8–53.1 |
| 48 weeks | 14 | 15.96 | 6.61 | 14.10 | 11.1–22.6 | 7.1–30.0 |
| 72 weeks | 15 | 13.37 | 4.23 | 12.00 | 10.3–16.4 | 8.6–22.2 |
| 96 weeks | 13 | 15.23 | 5.99 | 14.10 | 11.2–19.6 | 5.4–26.1 |
| 120 weeks | 12 | 14.53 | 5.52 | 12.15 | 11.0–15.9 | 10.0–29.4 |
| 144 weeks | 12 | 17.28 | 9.46 | 12.90 | 11.1–21.7 | 9.9–40.7 |
| Cr (mg/dL) |  |  |  |  |  |  |
| 0 week | 18 | 0.152 | 0.118 | 0.110 | 0.09–0.17 | 0.05–0.49 |
| 4 weeks | 18 | 0.179 | 0.134 | 0.120 | 0.08–0.24 | 0.05–0.48 |
| 12 weeks | 16 | 0.184 | 0.137 | 0.130 | 0.09–0.31 | 0.05–0.47 |
| 24 weeks | 16 | 0.168 | 0.166 | 0.095 | 0.07–0.18 | 0.05–0.60 |
| 48 weeks | 14 | 0.116 | 0.074 | 0.100 | 0.06–0.14 | 0.05–0.31 |
| 72 weeks | 15 | 0.114 | 0.078 | 0.100 | 0.07–0.14 | 0.03–0.35 |
| 96 weeks | 13 | 0.133 | 0.091 | 0.100 | 0.08–0.14 | 0.06–0.38 |
| 120 weeks | 12 | 0.130 | 0.085 | 0.100 | 0.07–0.16 | 0.05–0.33 |
| 144 weeks | 12 | 0.176 | 0.129 | 0.140 | 0.10–0.20 | 0.05–0.51 |
| UA (mg/dL) |  |  |  |  |  |  |
| 0 week | 18 | 5.32 | 1.25 | 5.45 | 4.4–6.4 | 2.3–7.0 |
| 4 weeks | 17 | 3.21 | 1.28 | 3.40 | 2.3–3.7 | 0.8–5.6 |
| 12 weeks | 16 | 3.64 | 1.87 | 3.55 | 2.3–4.2 | 1.1–8.9 |
| 24 weeks | 16 | 3.50 | 1.95 | 3.20 | 2.2–4.3 | 0.9–8.8 |
| 48 weeks | 14 | 2.91 | 0.98 | 2.70 | 2.3–3.7 | 0.9–4.4 |
| 72 weeks | 15 | 2.98 | 0.99 | 2.90 | 2.5–3.8 | 0.9–4.5 |
| 96 weeks | 13 | 3.26 | 1.44 | 2.90 | 2.4–4.1 | 0.8–6.3 |
| 120 weeks | 12 | 3.33 | 1.31 | 3.20 | 2.5–3.9 | 1.7–6.6 |
| 144 weeks | 12 | 3.42 | 1.37 | 3.50 | 2.3–4.3 | 1.5–6.2 |
| Cystatin C (mg/L) |  |  |  |  |  |  |
| 0 week | 18 | 0.899 | 0.237 | 0.825 | 0.79–1.05 | 0.61–1.51 |
| 4 weeks | 17 | 1.084 | 0.438 | 0.910 | 0.84–1.12 | 0.73–2.38 |
| 12 weeks | 15 | 1.075 | 0.530 | 0.910 | 0.81–1.13 | 0.67–2.80 |
| 20 weeks | 16 | 1.194 | 0.782 | 0.905 | 0.78–1.19 | 0.69–3.81 |
| 24 weeks | 16 | 1.029 | 0.409 | 0.920 | 0.74–1.16 | 0.66–2.30 |
| 28 weeks | 16 | 1.064 | 0.327 | 0.925 | 0.88–1.23 | 0.68–1.95 |
| 48 weeks | 15 | 0.973 | 0.304 | 0.890 | 0.81–1.14 | 0.58–1.77 |
| 72 weeks | 14 | 0.934 | 0.265 | 0.840 | 0.78–1.01 | 0.65–1.58 |
| 96 weeks | 13 | 0.988 | 0.295 | 0.910 | 0.83–1.01 | 0.68–1.63 |
| 120 weeks | 10 | 1.087 | 0.398 | 0.965 | 0.82–1.07 | 0.77–1.98 |
| 144 weeks | 12 | 1.155 | 0.572 | 0.985 | 0.81–1.09 | 0.75–2.57 |

Abbreviations: n: number, SD: standard deviation, IQR: interquartile range; BUN: blood urea nitrogen, Cr: creatinine, UA: uric acid

**Supplementary Table S6.** **MDQoL data.**

|  | Data n | Mean | SD | Median | IQR | Range | Change n | Mean | SD | Median | IQR | Range | 95% CI | P value* |
| --- | --- | --- | --- | --- | --- | --- | --- | --- | --- | --- | --- | --- | --- | --- |
| 1. Mental stability |  |  |  |  |  |  |  |  |  |  |  |  |  |  |
| 0 week | 17 | 52.6 | 16.1 | 53.1 | 38–63 | 31–88 |  |  |  |  |  |  |  |  |
| 24 weeks | 15 | 55.8 | 22.6 | 53.1 | 38–72 | 19–100 | 15 | 2.5 | 14.8 | 3.1 | -9–13 | -22–28 | -5.7 ～ 10.7 | 0.523 |
| 48 weeks | 13 | 57.0 | 16.5 | 56.3 | 50–69 | 25–88 | 13 | 2.6 | 16.7 | 3.1 | 0–6 | -41–25 | -7.5 ～ 12.7 |  |
| 96 weeks | 12 | 52.9 | 24.4 | 51.6 | 33–70 | 19–97 | 12 | -2.1 | 21.2 | 0.0 | -11–11 | -47–34 | -15.6 ～ 11.4 |  |
| 144 weeks | 11 | 59.4 | 23.5 | 56.3 | 47–81 | 13–88 | 11 | 3.4 | 23.3 | 12.5 | -6–16 | -53–25 | -12.2 ～ 19.0 |  |
| 2. ADL |  |  |  |  |  |  |  |  |  |  |  |  |  |  |
| 0 week | 17 | 49.5 | 29.8 | 43.8 | 28–69 | 0–100 |  |  |  |  |  |  |  |  |
| 24 weeks | 15 | 53.6 | 30.3 | 62.5 | 19–75 | 6–91 | 15 | -0.8 | 18.4 | -6.2 | -9–3 | -25–50 | -11.0 ～ 9.3 | 0.862 |
| 48 weeks | 13 | 47.4 | 31.5 | 56.3 | 22–63 | 0–100 | 13 | -2.6 | 14.5 | -6.3 | -9–0 | -19–25 | -11.4 ～ 6.1 |  |
| 96 weeks | 12 | 47.7 | 31.9 | 54.7 | 22–66 | 0–100 | 12 | -4.2 | 15.6 | -4.7 | -19–6 | -28–25 | -14.1 ～ 5.7 |  |
| 144 weeks | 11 | 48.0 | 24.7 | 46.9 | 31–63 | 13–100 | 11 | 0.0 | 19.6 | -6.2 | -16–13 | -25–47 | -13.2 ～ 13.1 |  |
| 3. Environment |  |  |  |  |  |  |  |  |  |  |  |  |  |  |
| 0 week | 17 | 71.6 | 17.1 | 69.4 | 61–83 | 44–100 |  |  |  |  |  |  |  |  |
| 24 weeks | 15 | 71.5 | 14.1 | 72.2 | 61–83 | 47–94 | 15 | -0.9 | 11.5 | -2.7 | -8–6 | -19–25 | -7.3 ～ 5.4 | 0.763 |
| 48 weeks | 13 | 68.4 | 18.1 | 69.4 | 53–86 | 39–94 | 13 | -1.5 | 12.3 | -5.5 | -11–8 | -17–22 | -8.9 ～ 5.9 |  |
| 96 weeks | 12 | 69.4 | 19.5 | 68.1 | 60–82 | 28–100 | 12 | -1.2 | 20.4 | 2.8 | -6–11 | -58–17 | -14.1 ～ 11.8 |  |
| 144 weeks | 11 | 69.2 | 21.5 | 75.0 | 50–89 | 39–100 | 11 | -2.0 | 19.6 | 0.0 | -17–11 | -47–22 | -15.2 ～ 11.2 |  |
| 3-1. Housing environment |  |  |  |  |  |  |  |  |  |  |  |  |  |  |
| 0 week | 17 | 71.6 | 17.1 | 69.4 | 61–83 | 44–100 |  |  |  |  |  |  |  |  |
| 24 weeks | 15 | 71.5 | 14.1 | 72.2 | 61–83 | 47–94 | 15 | -0.9 | 11.5 | -2.7 | -8–6 | -19–25 | -7.3 ～ 5.4 | 0.763 |
| 48 weeks | 13 | 68.4 | 18.1 | 69.4 | 53–86 | 39–94 | 13 | -1.5 | 12.3 | -5.5 | -11–8 | -17–22 | -8.9 ～ 5.9 |  |
| 96 weeks | 12 | 69.4 | 19.5 | 68.1 | 60–82 | 28–100 | 12 | -1.2 | 20.4 | 2.8 | -6–11 | -58–17 | -14.1 ～ 11.8 |  |
| 144 weeks | 11 | 69.2 | 21.5 | 75.0 | 50–89 | 39–100 | 11 | -2.0 | 19.6 | 0.0 | -17–11 | -47–22 | -15.2 ～ 11.2 |  |
| 3-2. Medical environment |  |  |  |  |  |  |  |  |  |  |  |  |  |  |
| 0 week | 17 | 71.6 | 17.1 | 69.4 | 61–83 | 44–100 |  |  |  |  |  |  |  |  |
| 24 weeks | 15 | 71.5 | 14.1 | 72.2 | 61–83 | 47–94 | 15 | -0.9 | 11.5 | -2.7 | -8–6 | -19–25 | -7.3 ～ 5.4 | 0.763 |
| 48 weeks | 13 | 68.4 | 18.1 | 69.4 | 53–86 | 39–94 | 13 | -1.5 | 12.3 | -5.5 | -11–8 | -17–22 | -8.9 ～ 5.9 |  |
| 96 weeks | 12 | 69.4 | 19.5 | 68.1 | 60–82 | 28–100 | 12 | -1.2 | 20.4 | 2.8 | -6–11 | -58–17 | -14.1 ～ 11.8 |  |
| 144 weeks | 11 | 69.2 | 21.5 | 75.0 | 50–89 | 39–100 | 11 | -2.0 | 19.6 | 0.0 | -17–11 | -47–22 | -15.2 ～ 11.2 |  |
| 3-3. Life environment |  |  |  |  |  |  |  |  |  |  |  |  |  |  |
| 0 week | 17 | 71.6 | 17.1 | 69.4 | 61–83 | 44–100 |  |  |  |  |  |  |  |  |
| 24 weeks | 15 | 71.5 | 14.1 | 72.2 | 61–83 | 47–94 | 15 | -0.9 | 11.5 | -2.7 | -8–6 | -19–25 | -7.3 ～ 5.4 | 0.763 |
| 48 weeks | 13 | 68.4 | 18.1 | 69.4 | 53–86 | 39–94 | 13 | -1.5 | 12.3 | -5.5 | -11–8 | -17–22 | -8.9 ～ 5.9 |  |
| 96 weeks | 12 | 69.4 | 19.5 | 68.1 | 60–82 | 28–100 | 12 | -1.2 | 20.4 | 2.8 | -6–11 | -58–17 | -14.1 ～ 11.8 |  |
| 144 weeks | 11 | 69.2 | 21.5 | 75.0 | 50–89 | 39–100 | 11 | -2.0 | 19.6 | 0.0 | -17–11 | -47–22 | -15.2 ～ 11.2 |  |
| 4. Hope |  |  |  |  |  |  |  |  |  |  |  |  |  |  |
| 0 week | 17 | 61.8 | 22.7 | 67.9 | 46–82 | 14–86 |  |  |  |  |  |  |  |  |
| 24 weeks | 15 | 55.5 | 18.9 | 60.7 | 39–71 | 11–75 | 15 | -6.2 | 24.4 | -7.2 | -14–11 | -75–39 | -19.7 ～ 7.3 | 0.343 |
| 48 weeks | 13 | 50.0 | 20.6 | 50.0 | 39–64 | 14–86 | 13 | -9.3 | 20.6 | -3.5 | -14–4 | -71–7 | -21.8 ～ 3.1 |  |
| 96 weeks | 12 | 60.1 | 23.4 | 67.9 | 41–75 | 7–86 | 12 | -0.9 | 30.5 | 1.8 | -9–9 | -79–57 | -20.3 ～ 18.5 |  |
| 144 weeks | 11 | 60.0 | 16.9 | 57.1 | 46–71 | 29–86 | 11 | 0.6 | 29.9 | 7.1 | -29–14 | -57–57 | -19.4 ～ 20.7 |  |
| 5. Activity |  |  |  |  |  |  |  |  |  |  |  |  |  |  |
| 0 week | 17 | 42.4 | 30.4 | 45.0 | 20–60 | 0–100 |  |  |  |  |  |  |  |  |
| 24 weeks | 15 | 51.3 | 28.1 | 55.0 | 25–70 | 0–100 | 15 | 7.0 | 34.2 | 0.0 | -5–20 | -45–100 | -11.9 ～ 25.9 | 0.441 |
| 48 weeks | 13 | 51.2 | 28.1 | 45.0 | 30–60 | 10–100 | 13 | 6.5 | 28.2 | 10.0 | 0–20 | -55–55 | -10.5 ～ 23.6 |  |
| 96 weeks | 12 | 43.3 | 28.5 | 40.0 | 30–58 | 0–100 | 12 | -3.8 | 28.9 | -2.5 | -15–20 | -60–30 | -22.1 ～ 14.6 |  |
| 144 weeks | 11 | 49.5 | 31.4 | 50.0 | 20–80 | 5–100 | 11 | 0.0 | 42.4 | -5.0 | -30–20 | -55–80 | -28.5 ～ 28.5 |  |
| 6. Sense of well-being |  |  |  |  |  |  |  |  |  |  |  |  |  |  |
| 0 week | 17 | 63.2 | 31.6 | 75.0 | 33–92 | 17–100 |  |  |  |  |  |  |  |  |
| 24 weeks | 15 | 58.9 | 25.3 | 66.7 | 42–83 | 8–83 | 15 | -8.3 | 33.0 | -8.4 | -33–17 | -67–50 | -26.6 ～ 9.9 | 0.344 |
| 48 weeks | 13 | 57.1 | 31.9 | 50.0 | 42–75 | 0–100 | 13 | -12.2 | 32.0 | -8.3 | -33–0 | -67–42 | -31.5 ～ 7.2 |  |
| 96 weeks | 12 | 62.5 | 34.6 | 66.7 | 33–96 | 8–100 | 12 | -9.0 | 34.0 | -4.2 | -21–0 | -67–67 | -30.6 ～ 12.6 |  |
| 144 weeks | 11 | 71.2 | 30.4 | 75.0 | 33–100 | 25–100 | 11 | 2.3 | 19.4 | 0.0 | 0–17 | -42–33 | -10.8 ～ 15.3 |  |
| 7. Human relationship |  |  |  |  |  |  |  |  |  |  |  |  |  |  |
| 0 week | 17 | 63.2 | 21.4 | 65.0 | 50–75 | 25–100 |  |  |  |  |  |  |  |  |
| 24 weeks | 15 | 58.3 | 23.4 | 50.0 | 40–80 | 30–100 | 15 | -5.7 | 20.3 | -5.0 | -20–15 | -40–25 | -16.9 ～ 5.5 | 0.297 |
| 48 weeks | 13 | 58.1 | 23.3 | 60.0 | 45–75 | 20–100 | 13 | -5.0 | 20.8 | -5.0 | -10–0 | -50–45 | -17.6 ～ 7.6 |  |
| 96 weeks | 12 | 65.0 | 18.7 | 67.5 | 50–80 | 30–90 | 12 | 3.3 | 21.6 | 0.0 | -8–5 | -20–60 | -10.4 ～ 17.0 |  |
| 144 weeks | 11 | 62.7 | 19.8 | 60.0 | 45–80 | 35–100 | 11 | -2.3 | 14.9 | 0.0 | -20–5 | -20–30 | -12.3 ～ 7.7 |  |
| 8. Family |  |  |  |  |  |  |  |  |  |  |  |  |  |  |
| 0 week | 17 | 72.6 | 21.1 | 80.0 | 55–90 | 35–100 |  |  |  |  |  |  |  |  |
| 24 weeks | 15 | 73.0 | 20.6 | 70.0 | 60–90 | 40–100 | 15 | 1.0 | 13.4 | 0.0 | 0–10 | -25–25 | -6.4 ～ 8.4 | 0.777 |
| 48 weeks | 13 | 68.8 | 22.3 | 60.0 | 50–90 | 45–100 | 13 | -2.3 | 14.2 | 0.0 | -10–10 | -35–15 | -10.9 ～ 6.3 |  |
| 96 weeks | 12 | 77.1 | 18.9 | 77.5 | 60–95 | 50–100 | 12 | 6.7 | 12.9 | 0.0 | 0–15 | -10–35 | -1.5 ～ 14.8 |  |
| 144 weeks | 11 | 72.3 | 22.8 | 65.0 | 60–100 | 30–100 | 11 | 3.6 | 14.0 | 0.0 | -5–15 | -20–25 | -5.8 ～ 13.0 |  |
| 9. Sex |  |  |  |  |  |  |  |  |  |  |  |  |  |  |
| 0 week | 17 | 64.2 | 28.4 | 66.7 | 42–83 | 17–100 |  |  |  |  |  |  |  |  |
| 24 weeks | 15 | 72.2 | 20.3 | 66.7 | 58–100 | 42–100 | 15 | 9.4 | 17.2 | 16.6 | 0–25 | -25–33 | -0.1 ～ 19.0 | 0.052 |
| 48 weeks | 13 | 75.6 | 17.5 | 75.0 | 67–83 | 42–100 | 13 | 14.7 | 19.9 | 16.7 | 0–25 | -25–42 | 2.7 ～ 26.8 |  |
| 96 weeks | 12 | 72.9 | 27.3 | 70.9 | 58–100 | 25–100 | 12 | 12.5 | 30.7 | 12.5 | 0–25 | -42–67 | -7.0 ～ 32.0 |  |
| 144 weeks | 11 | 71.2 | 29.2 | 75.0 | 58–100 | 0–100 | 11 | 14.4 | 41.5 | 25.0 | 0–33 | -100–50 | -13.5 ～ 42.3 |  |
| 10. Respiration and swallowing function |  |  |  |  |  |  |  |  |  |  |  |  |  |  |
| 0 week | 17 | 58.5 | 18.8 | 60.0 | 45–65 | 30–100 |  |  |  |  |  |  |  |  |
| 24 weeks | 15 | 61.7 | 31.0 | 65.0 | 35–90 | 0–100 | 15 | 0.0 | 24.7 | 0.0 | -20–20 | -40–50 | -13.7 ～ 13.7 | 1.000 |
| 48 weeks | 13 | 62.3 | 29.1 | 65.0 | 40–80 | 15–100 | 13 | 1.5 | 28.1 | 0.0 | -15–10 | -50–50 | -15.4 ～ 18.5 |  |
| 96 weeks | 12 | 59.2 | 29.8 | 60.0 | 30–85 | 15–100 | 12 | -1.3 | 21.1 | 0.0 | -23–15 | -30–30 | -14.7 ～ 12.2 |  |
| 144 weeks | 11 | 60.5 | 26.9 | 55.0 | 45–90 | 25–100 | 11 | 0.0 | 11.6 | 0.0 | -10–10 | -15–15 | -7.8 ～ 7.8 |  |
| 11. Bowel movement |  |  |  |  |  |  |  |  |  |  |  |  |  |  |
| 0 week | 17 | 64.7 | 24.7 | 62.5 | 50–88 | 25–100 |  |  |  |  |  |  |  |  |
| 24 weeks | 15 | 65.0 | 35.4 | 50.0 | 25–100 | 13–100 | 15 | 0.8 | 30.8 | 0.0 | -13–25 | -63–50 | -16.2 ～ 17.9 | 0.918 |
| 48 weeks | 13 | 70.2 | 33.3 | 87.5 | 38–100 | 25–100 | 13 | 7.7 | 29.1 | 0.0 | -13–38 | -50–50 | -9.9 ～ 25.3 |  |
| 96 weeks | 12 | 59.4 | 34.2 | 50.0 | 38–100 | 0–100 | 12 | -6.3 | 32.6 | 0.0 | -19–13 | -75–38 | -27.0 ～ 14.5 |  |
| 144 weeks | 11 | 62.5 | 23.0 | 62.5 | 50–75 | 25–100 | 11 | -4.5 | 27.5 | 0.0 | -25–13 | -50–38 | -23.0 ～ 14.0 |  |

Abbreviations: n: number, SD: standard deviation, IQR: interquartile range, CI: confidence interval; MDQoL: Muscular dystrophy quality of life-60, ADL: Activities of daily living

**Supplementary Table S7.** **SF-12 data.**

|  | Data n | Mean | SD | Median | IQR | Range | Change n | Mean | SD | Median | IQR | Range | 95% CI | P value* |
| --- | --- | --- | --- | --- | --- | --- | --- | --- | --- | --- | --- | --- | --- | --- |
| Physical functioning |  |  |  |  |  |  |  |  |  |  |  |  |  |  |
| 0 week | 17 | 9.0 | 14.1 | 2.7 | 3–3 | 3–56 |  |  |  |  |  |  |  |  |
| 24 weeks | 14 | 5.6 | 5.6 | 2.7 | 3–3 | 3–16 | 14 | -4.7 | 16.9 | 0.0 | 0–0 | -53–13 | -14.5 ～ 5.0 | 0.315 |
| 48 weeks | 12 | 8.2 | 15.4 | 2.7 | 3–3 | 3–56 | 12 | -2.2 | 9.5 | 0.0 | 0–0 | -27–13 | -8.3 ～ 3.8 |  |
| 96 weeks | 12 | 4.9 | 5.2 | 2.7 | 3–3 | 3–16 | 12 | -5.5 | 18.3 | 0.0 | -7–0 | -53–13 | -17.1 ～ 6.1 |  |
| 144 weeks | 11 | 8.7 | 16.1 | 2.7 | 3–3 | 3–56 | 11 | -2.4 | 22.8 | 0.0 | 0–0 | -53–40 | -17.7 ～ 12.9 |  |
| Role physical |  |  |  |  |  |  |  |  |  |  |  |  |  |  |
| 0 week | 17 | 26.1 | 18.9 | 30.2 | 11–30 | 5–56 |  |  |  |  |  |  |  |  |
| 24 weeks | 15 | 29.8 | 18.8 | 30.2 | 17–56 | 5–56 | 15 | 0.9 | 21.4 | 6.4 | -13–13 | -51–26 | -11.0 ～ 12.7 | 0.880 |
| 48 weeks | 13 | 40.6 | 19.3 | 49.4 | 24–56 | 5–56 | 13 | 9.3 | 28.2 | 12.8 | 0–26 | -45–51 | -7.7 ～ 26.4 |  |
| 96 weeks | 12 | 36.6 | 17.7 | 43.0 | 27–49 | 5–56 | 12 | 3.2 | 24.9 | 6.4 | -6–16 | -51–45 | -12.6 ～ 19.0 |  |
| 144 weeks | 11 | 34.3 | 19.4 | 30.2 | 17–56 | 5–56 | 11 | 0.6 | 25.8 | 0.0 | -26–26 | -26–45 | -16.7 ～ 17.9 |  |
| Bodily pain |  |  |  |  |  |  |  |  |  |  |  |  |  |  |
| 0 week | 17 | 49.4 | 11.7 | 57.3 | 35–57 | 24–57 |  |  |  |  |  |  |  |  |
| 24 weeks | 15 | 48.4 | 12.1 | 57.3 | 35–57 | 24–57 | 15 | -3.7 | 11.7 | 0.0 | -11–0 | -22–22 | -10.2 ～ 2.7 | 0.238 |
| 48 weeks | 13 | 47.0 | 16.7 | 57.3 | 46–57 | 13–57 | 13 | -6.0 | 16.1 | 0.0 | -11–0 | -45–22 | -15.8 ～ 3.8 |  |
| 96 weeks | 12 | 47.1 | 15.4 | 57.3 | 41–57 | 13–57 | 12 | -5.6 | 16.1 | 0.0 | -11–0 | -45–22 | -15.8 ～ 4.7 |  |
| 144 weeks | 11 | 43.1 | 17.3 | 57.3 | 24–57 | 13–57 | 11 | -9.1 | 19.2 | 0.0 | -22–0 | -45–22 | -22.0 ～ 3.8 |  |
| General health |  |  |  |  |  |  |  |  |  |  |  |  |  |  |
| 0 week | 15 | 51.6 | 5.3 | 51.9 | 52–52 | 36–64 |  |  |  |  |  |  |  |  |
| 24 weeks | 15 | 54.7 | 7.7 | 51.9 | 52–64 | 36–64 | 15 | 3.1 | 9.6 | 0.0 | 0–12 | -16–28 | -2.2 ～ 8.4 | 0.234 |
| 48 weeks | 13 | 51.7 | 13.8 | 51.9 | 52–64 | 24–70 | 13 | -1.1 | 12.9 | 0.0 | 0–7 | -28–12 | -8.8 ～ 6.7 |  |
| 96 weeks | 12 | 50.2 | 7.5 | 51.9 | 52–52 | 36–64 | 12 | -2.7 | 8.0 | 0.0 | -6–0 | -16–12 | -7.8 ～ 2.4 |  |
| 144 weeks | 11 | 51.5 | 6.3 | 51.9 | 52–52 | 36–64 | 11 | -1.5 | 7.1 | 0.0 | 0–0 | -16–12 | -6.3 ～ 3.3 |  |
| Vitality |  |  |  |  |  |  |  |  |  |  |  |  |  |  |
| 0 week | 17 | 42.2 | 11.1 | 38.5 | 39–48 | 29–66 |  |  |  |  |  |  |  |  |
| 24 weeks | 15 | 45.1 | 8.7 | 47.6 | 39–48 | 29–57 | 15 | 2.4 | 11.1 | 0.0 | 0–9 | -18–18 | -3.7 ～ 8.6 | 0.413 |
| 48 weeks | 13 | 46.9 | 12.0 | 47.6 | 48–48 | 29–66 | 13 | 4.2 | 13.1 | 9.1 | 0–18 | -18–18 | -3.8 ～ 12.1 |  |
| 96 weeks | 12 | 43.8 | 12.5 | 47.6 | 29–52 | 29–66 | 12 | 0.8 | 10.6 | 0.0 | 0–9 | -18–18 | -6.0 ～ 7.5 |  |
| 144 weeks | 11 | 40.1 | 12.7 | 38.5 | 29–48 | 29–66 | 11 | -2.5 | 13.5 | 0.0 | -18–9 | -27–18 | -11.5 ～ 6.6 |  |
| Social functioning |  |  |  |  |  |  |  |  |  |  |  |  |  |  |
| 0 week | 17 | 46.5 | 12.1 | 56.6 | 34–57 | 22–57 |  |  |  |  |  |  |  |  |
| 24 weeks | 15 | 43.6 | 16.1 | 45.1 | 34–57 | 11–57 | 15 | -5.3 | 15.5 | 0.0 | -23–0 | -23–23 | -13.9 ～ 3.3 | 0.204 |
| 48 weeks | 13 | 44.3 | 18.4 | 56.6 | 34–57 | 11–57 | 13 | -6.2 | 19.6 | 0.0 | -11–0 | -46–23 | -18.0 ～ 5.7 |  |
| 96 weeks | 12 | 41.3 | 22.5 | 56.6 | 11–57 | 11–57 | 12 | -8.6 | 21.3 | 0.0 | -23–0 | -46–23 | -22.2 ～ 5.0 |  |
| 144 weeks | 11 | 42.0 | 19.2 | 56.6 | 22–57 | 11–57 | 11 | -7.3 | 22.5 | 0.0 | -23–11 | -46–23 | -22.4 ～ 7.8 |  |
| Role emotional |  |  |  |  |  |  |  |  |  |  |  |  |  |  |
| 0 week | 17 | 49.5 | 9.3 | 56.3 | 44–56 | 32–56 |  |  |  |  |  |  |  |  |
| 24 weeks | 15 | 38.9 | 14.8 | 38.1 | 32–56 | 8–56 | 15 | -9.7 | 14.1 | -6.1 | -12–0 | -49–6 | -17.5 ～ -1.9 | 0.018 |
| 48 weeks | 13 | 43.2 | 15.8 | 44.1 | 32–56 | 8–56 | 13 | -7.0 | 17.8 | 0.0 | -12–0 | -49–24 | -17.8 ～ 3.8 |  |
| 96 weeks | 12 | 44.6 | 15.4 | 50.2 | 38–56 | 8–56 | 12 | -5.1 | 17.5 | 0.0 | -12–3 | -49–24 | -16.2 ～ 6.1 |  |
| 144 weeks | 11 | 46.3 | 11.9 | 56.3 | 32–56 | 32–56 | 11 | -2.8 | 15.4 | 0.0 | -18–0 | -24–24 | -13.1 ～ 7.6 |  |
| Mental health |  |  |  |  |  |  |  |  |  |  |  |  |  |  |
| 0 week | 17 | 51.4 | 6.9 | 51.8 | 46–52 | 40–64 |  |  |  |  |  |  |  |  |
| 24 weeks | 15 | 47.8 | 11.5 | 45.8 | 40–58 | 28–64 | 15 | -3.6 | 10.9 | 0.0 | -12–6 | -24–12 | -9.6 ～ 2.4 | 0.219 |
| 48 weeks | 13 | 45.3 | 14.8 | 45.8 | 40–58 | 16–64 | 13 | -6.5 | 14.4 | -6.0 | -12–0 | -36–12 | -15.2 ～ 2.2 |  |
| 96 weeks | 12 | 48.3 | 13.2 | 51.8 | 40–58 | 16–64 | 12 | -3.0 | 11.9 | 0.0 | -9–6 | -24–12 | -10.6 ～ 4.5 |  |
| 144 weeks | 11 | 47.4 | 11.8 | 45.8 | 40–58 | 22–64 | 11 | -4.9 | 13.1 | -6.0 | -18–6 | -24–12 | -13.7 ～ 3.9 |  |

Abbreviations: n: number, SD: standard deviation, IQR: interquartile range, CI: confidence interval
